# Hanwell Asylum
*The Eighth Report of the Committee of Visitors of the County Lunatic Asylum at Hanwell. London. 1853.


**Published:** 1853-07-01

**Authors:** 


### HANWELL ASYLUM.*

Any Report of Hanwell Asylum will command a degree of attention which that of no similar institution can yet obtain. This will be recognised as due to the circumstance of that asylum having been the field for carrying out and illustrating the practicability of the so-called "non-restraint" system of treating the insane. Hence every matter touching this asylum has been treated with indulgence, if not with a species of veneration. Yet at the present day, Hanwell both in construction and organization will, if examined, not bear comparison with many other asylums. Its halcyon days are past, and no one conversant with the state of other kindred institutions will vaunt it now as the great model asylum of England. Yet such it was at one period considered.

The present managers of Hanwell appear still to regard it as perfect in all its arrangements, and an example of what an asylum should be. They are proud of its great antecedents, and feel elevated by the halo of glory surrounding it. If we are to believe the present report, the asylum is indebted for its reputation, not to the *medical officers*, but to the Committee of Management. The Chairman, Mr. Wilks, has done his utmost to impress this upon our minds. He says,—"Your *committee* have laboured to perform their arduous duties with perseverance and zeal, and, convinced of the peculiar qualities demanded, they have endeavoured to combine firmness with care, and discretion with kindness of heart; and they trust that they have been enabled to promote the welfare and comfort of the numerous and hapless beings committed to their charge." And "your committee would express their deep and devout gratitude for the triumphant success that has attended on *their remedial measures*, (!) and on the entire absence of all cruelty, coercion, or needless restraint, from their free, contented, and cheerful asylum."

Our readers must feel grateful for this new light, if, like ourselves, they in their blindness, thought patients were under the charge of *medical* officers, whose duty it was to apply remedial measures, and to direct the moral management of the Institution; and of whom the peculiar qualities were demanded, firmness mingled with kindness and discretion.

Moreover, aided by this light, we can understand the rule laid down with respect to the office of consulting-physician, whose opinion is to be sought in any cases which may appear to the *committee* to require such further advice!

In recognising, therefore, the all-importance of the committee's duties, we may be allowed to join in the expression of satisfaction and gratitude for "the triumphant success which has attended *their* labours," "aided by the staff of officers," and we would fain hope that by their continued assiduity, the members of the committee may so perfect themselves in the treatment and care of lunatics, that they will, by another year, no longer require to be "aided," least of all, by medical officers.

If further proof were needed of the importance of the visiting justices, and the non-importance of the resident medical officers, it might be furnished by reference to the space and position allowed to the reports of the latter, which follow after the "returns of the excellent matron:" but of these more hereafter."

To return to the matter and manner of the chairman's report. As the glorification of the committee alone could not be so "long drawn out" as to

* The Eighth Report of the Committee of Visitors of the County Lunatic Asylum at Hanwell. London. 1853.

occupy five-and-twenty pages, Mr. Wilks has had recourse to various means of expansion to satisfy his *cacoethes scribendi.* Thus three pages are occupied by a list of 'works,' 'minute details' of trifling expenditure for repairs of 'rocking-horses,' papering rooms, altering water-closets, &c., &c.; two pages of particulars from the "indefatigable and judicious storekeeper," concerning work done, condition of live stock, bedding and clothing, which are called "lucid and demonstrative details;" and then follow sundry pages in which are reproduced much of the reports of the various officers of the establishment, which are printed at length in another part of the pamphlet. In page seventeen, an extension of space is ingeniously gained by six figures being made to do duty for six lines—a device deserving the attention of all penny-a-liners.

The worthy chairman's 'conclusion' occupies three pages; to it he has more particularly reserved the pathos of his writing. For pathos he must display, lest comparison might be odious, seeing that he discovers the matron's report replete with that element of composition. It is developed by contrasts, like the vivid impression of a white skin after beholding a "nigger." We have an "auspicious aspect" of a "free, (?) contented, and cheerful asylum, contrasted with bolts, bars, and dungeons; with "intemperate and infuriate attendants, conjoined with instruments of torture worthy only of the days of the bloody inquisition." Then we are told of what might have once been observed; the story "of a very clever lady, the mistress of many languages," neglected and chained; and, finally, that of the iron-bound man of Bethlem notoriety in years gone by; with these are made appear, in flattering comparison, "the quiet and smiling groups of clean and tidy women;" "the active, energetic agricultural labourers, with willing step and stalwart frame."

Now, if there had been anything original in this grandiloquent description, if it had not been much more than a twice told tale, we might probably have somewhat appreciated the author's rhetoric; but in its present character it savours of clap-trap. For to obtain his favourable contrast, the writer goes too far back; he rakes up bygone events now deplored by all; he courts applause, like those who endeavour to obtain character by referring to the mal-practices and imperfect civilization of a past generation.

It would have been fair if Mr. Wilks had contrasted Hanwell, *as it is*, with other county asylums, and to have shown its superior general medical organization. Let the chairman attempt this next year.

The style of the Report is verbose, diffuse, and inflated. Besides, what quotations elsewhere introduced may furnish, we might present, did space permit, many curious, if not amusing examples. To instance a few: we read of "industry cultivated with exemplary diligence;" of banquets which "have formed most important items in the economies of comforts and health." But we are quite puzzled at the combination of emotions which Mr. Wilks tells us should be excited by the narrative of the "Bethlem case;" and we must place it before our readers for explanation. He says it was "a case of such atrocity, that your committee record it to excite at once approval, and the liveliest gratitude, disgust, and true delight, at the new aspect of affairs around them." Now, it does seem a difficulty to us to entertain disgust and true delight, at one and the same time, in respect of the same object; but we are open to conviction.

There are some circumstances spoken of in the Report not to be overlooked in our remarks. We would especially notice the discontinuance of the schoolmaster. His appointment was, the other day, considered as a matter of great exultation by the magistrates, and held forth as an example to be followed by other lunatic asylums. We are now informed, that his services for the future "will be unnecessary." We cannot understand this proceeding. The appointment of such an officer is becoming more and more recognised as desirable, and its benefits daily more patent. Moreover, Mr. Waite, the late schoolmaster at Hanwell, reports favourably, speaks of considerably increased numbers attending the school, of the prevalent desire to attend it, of the interest exhibited in the lectures, &c., given by him. Were these benefits obvious to the medical officers? Did they witness no cases benefited by the schoolmaster's instruction? Did they advise, or were they even consulted in the abolition of the office? we should like these questions answered, for we can scarcely believe this step can have been taken with the acquiescence of those gentlemen. But we are reminded, if we are to believe the professions of the committee, that the medical officers are but ciphers in the treatment of the patients, and that, therefore, it is not to be supposed their opinion was sought for in the matter.

The committee assign no valid reason for dispensing with the services of the schoolmaster, unless the saving of a few pounds a year is to be considered as an equivalent. This pecuniary gain, Mr. Wilks says, "calls for attention and *approval*." We think it calls rather for attention and *censure*, a result it is more likely to obtain.

We read, p. 18—"The duties of the dispenser have also been increased, so as to obviate the supposed necessity for a general superintendence of the male division."

From this observation it would appear that an additional medical officer is required for the male side of the asylum; and when it is understood that there are 411 male patients to be attended to, such an increase of the staff must, to most men, appear imperatively demanded. But to the infallible committee it is but a "supposed necessity," and they impose additional duties on an officer, whom, we should think, had more than enough to attend to in his own department, in the preparation of medicines, and in superintending their proper administration, as well as in the keeping the various case-books, &c.

We regret to see that the active committee of this asylum does not appreciate the necessity of appointing an additional medical officer on the female side, where 552 patients are to be morally and medically treated by one individual!

We are convinced, however, that as public opinion becomes more enlightened, and when the important fact is recognised that the cure and not the mere care of the insane is the great object, even upon economic grounds, visiting magistrates of asylums will be compelled to augment their medical staff, and that the imperative need of such increase will not be able to be ignored as a "supposed necessity."

Year by year we witness the erection of new county asylums, of old ones enormously enlarged, and of the congregation of the insane by hundreds within them; yet the cry is, "Still they come," and no room can be found for the admission of recent cases. Such is the present system: hopeful, recent cases are excluded; are allowed to remain in ill-adapted workhouse wards, or are altogether uncared for, and when they become hopeless and incurable, they are admitted, and assist to swell the insane population of our over-grown asylums.

But allowing that a case of recent origin does find its way into one of our gigantic county asylums, what attention and treatment can be given to it? Will it not, necessarily, be imperfect? The case supposed may probably be one of 500 assigned to the care of one medical man; it can rightly demand only its proportion of attention; the 499 other patients are to be supervised medically, to have their histories known, the phases of their malady understood, their mental aberrations controlled and directed, their moral discipline regulated, their industrial tendencies evoked, and, in fine, to be generally superintended in those multitudinous details which devolve on the medical director of an asylum.

The case in point may indeed be one of great solicitude, and may obtain the special attention and treatment it naturally demands; but this is accomplished at the cost of the other patients. For the requirements of even the half of the number of lunatics supposed, cannot possibly be attended to justly by any one man of the ordinary human mould, having only a certain tether, whether physical, moral, or intellectual. Hence, most surely, there must be inefficient treatment: patients cannot have that care devoted to them which they ought, or that which justice demands, viewed alone and without reference to the requirements of that highest gift of charity—love to the afflicted, which is above all price, and which should actuate and prompt us all to the exercise of that golden rule, "to do to others as we would they should do unto us."

The effects of the system in question are pernicious in every way. The medical superintendent feels the impossibility of doing his duty as conscience indicates: he is obliged to rest content with the observance of the printed rules laid down for his guidance; and, we will venture to remark, with all respect to the feelings of this meritorious class of public officer,—he is ever prone to degenerate into routine, and to allow his special medical knowledge to succumb under the pressure of the general duties of management. What may be effected under another state of things, when opportunity of treatment is afforded in an early stage of the malady, may be seen by reference to the records of such institutions as Bethlem and St. Luke's. Under the workings of the special rules of those hospitals framed to exclude old and hopeless cases, evaded, though those rules frequently are, 70 per cent. and upwards of the insane are cured.

This fact should certainly be borne in mind by country magistrates. If, from the accumulation of chronic cases, large asylums, or "Refuges" for such are needed, other provisions should be made for recent instances of insanity. There should be asylums or hospitals for county patients, moderate in size, and having a sufficient staff of medical officers to afford the requisite time and attention to the treatment of individuals. By such a system what happy results would follow? How many might be returned to their homes to gain their own livelihood, instead of, as now, becoming permanently chargeable upon their parishes.

We must now briefly advert to other reports printed with the chairman's. The next in length is that of the matron, which has called forth so lively an admiration from Mr. Wilks, as "replete with interest and pathos;" it is accordingly "presented in an unmutilated form." It exhibits an attempt at fine writing, more pardonable than in the case of the chairman, and less in extent and degree. It contains nothing worth extracting, being made up of notices of some few female patients, and an account of entertainments given. However, we must state our opinion, that a matron's printed report is uncalled for. If cases occur of sufficient interest to be detailed, it seems to us that the medical officers are the most fitting persons to narrate them.

As Mr. Wilks has taken the lion's share of space for his report, consisting of only twenty-five pages, and as *eight* have been allotted to the matron, the medical officers cannot be allowed to occupy much room with their lucubrations. Consequently Dr. Begley is cramped in about *jour*, and Mr. Denne is obliged to be content with *two* pages! Of the space allowed him, Dr. Begley, superintending the male department, has made the best use. Most of his observations apply to general paralysis, and are highly valuable; our only regret is that they are not more extended, so as to represent to us the results of his great and lengthened experience at Hanwell, and the almost unequalled opportunities of collecting facts regarding that dire, though interesting malady.

Our space forbids quotations, but we may note that Dr. Begley shows that life is often prolonged in general paralysis "much beyond the period assigned as its limit by continental writers, who give three years as its ultimate duration;" and he refers to cases falling under his own observation which have lasted from five to eight, and even to twelve years.

We recommend Dr. Begley's observations on this disease to our reader's attention.

In the course of the year, one man committed suicide by precipitating himself into an area twelve feet deep, and thereby fracturing his skull and cervical vertebræ.

There is a distressing fact stated by Dr. Begley, which claims consideration —viz., that of the 963 patients now in the asylum, nearly all are incurable; that "the possibly curable do not exceed fifteen in number, and of these, probably not more than ten will recover." We fear, too, that a similar statement might be made in regard to Colney Hatch Asylum, with its 1100 inmates; and besides all those in the two county asylums, there are some hundreds of pauper patients, mostly hopelessly incurable, belonging to Middlesex, distributed in workhouses and private asylums.

Knowing these facts, we may well enquire when will asylum accommodation be found for all the lunatics chargeable to this county, especially if the present system be persevered in?

In the report of Mr. Denne, the medical officer on the female side, only one particular offers itself for notice. It is that of the suicide of a female, who, "whilst sitting at dinner, thrust the metal fork down her throat, after which she lived thirteen days." We are not told the immediate cause of death.

Besides the returns noticed, there are those from the chaplain and the late schooolmaster. Suffice it to say, they are satisfactory and gratifying.

It has by this time, doubtless, become generally known that Dr. Conolly resigned the appointment of visiting physician of Hanwell, last year (1852). Desiring still to retain some connexion with the institution, so intimately associated with his name and reputation, he solicited the honorary appointment of consulting physician, which was agreed to unanimously by the committee of visitors.

In conclusion, we would recommend the committee, and Mr. Wilks, as their exponent, to eschew in the report of Hanwell, for the future, parading their own zeal, ability, discretion, and importance in the management of the unfortunate inmates of the asylum; to content themselves with making the due returns of receipts and expenditure, and of kindred matters; and so to leave their medical officers to report on medical subjects, on the health of the patients, the means of treatment, the effects of employment, &c. Moreover, if the committee would do their duty as general supervisors for the interests of the rate-payers, let them not fail to recognise the medical officers as the chief agents in the working of the institution; let them encourage those servants in their arduous duties, by duly and adequately recognising the value of their services; and let the committee no longer omit to afford them that additional medical aid which is absolutely necessary both to the interests of those who have to maintain the asyl.im, and to the treatment of those whose dreadful malady unfortunately compels them to be the inmates.

---

